# Enhancing Communication in Dementia Care: Raising the Role of Augmentative and Alternative Communication (AAC)

**DOI:** 10.1093/geroni/igaf122.421

**Published:** 2025-12-31

**Authors:** Jing Wang, Chloe Mitchell, Aubrie Woodward, Kay Chen

**Affiliations:** University of New Hampshire, Durham, New Hampshire, United States; University of New Hampshire, Durham, New Hampshire, United States; University of New Hampshire, Durham, New Hampshire, United States; University of New Hampshire, Durham, New Hampshire, United States

## Abstract

Effective communication is fundamental to maintaining quality of life and person-centered care for individuals with dementia. However, cognitive decline progressively impairs verbal expression and comprehension, leading to social withdrawal, frustration, and increased caregiver burden. Augmentative and Alternative Communication (AAC) interventions offer promising strategies to support communication in dementia care, yet their integration into routine practice remains limited. This narrative review synthesizes current research on AAC interventions for dementia, identifies key gaps, and outlines future directions to enhance their effectiveness and implementation. We conducted a narrative review of peer-reviewed studies, reports, and guidelines on AAC in dementia care, focusing on intervention types, efficacy, and integration into care practices. Findings indicate that AAC strategies range from using low-tech solutions (e.g., memory books, visual cues, and communication boards) to high-tech system (e.g., speech-generating devices and mobile applications). While research suggests AAC can improve engagement, decision-making, and social participation, its long-term effectiveness and real-world application remain underexplored. Major challenges include the need for personalization based on disease progression, cultural considerations, and scalable caregiver training. Additionally, limited research exists on how AAC can be effectively embedded into standard dementia care workflows. Addressing these gaps is essential for optimizing AAC interventions and ensuring their accessibility and sustainability. Future research should prioritize longitudinal studies to assess sustained impact, strategies for culturally responsive adaptation, and scalable caregiver training models. Advancing AAC in dementia care has the potential to enhance person-centered communication, facilitate shared decision-making, and improve quality of life for individuals with dementia and their caregivers.

